# *S*-4-Chloro­phenyl 9,10-dihydro­acridine-9-carbothio­ate

**DOI:** 10.1107/S1600536809003468

**Published:** 2009-02-04

**Authors:** Yanbing Duan, Haifeng Sun, Peng Guo, Min Ji

**Affiliations:** a School of Chemistry and Chemical Engineering, Southeast University, 210096 Nanjing, People’s Republic of China; b Changchun Institute of Applied Chemistry, ChineseAcademy of Sciences, 130022 Changchun, People’s Republic of China

## Abstract

In tricyclic fragment of the title mol­ecule, C_20_H_14_ClNOS, the central 1,4-dihydro­pyridine ring adopts a boat conformation while the two benzene rings form a dihedral angle of 17.38 (5)°. In the crystal structure, weak inter­molecular N—H⋯O hydrogen bonds link the mol­ecules into chains propagating along the *b* axis.

## Related literature

For applications of acridine derivatives, see: Dodeigne *et al.* (2000 ▶); Ashmore *et al.* (2008 ▶); Zomer & Jacquemijns (2001 ▶).
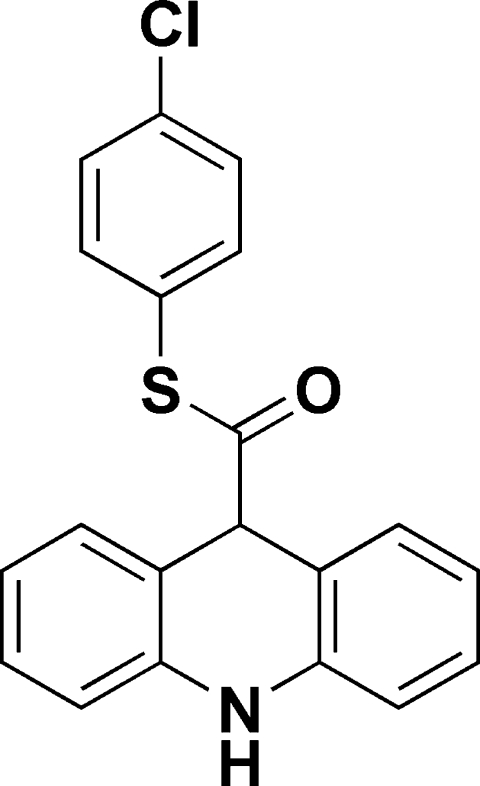


## Experimental

### 

#### Crystal data

C_20_H_14_ClNOS
*M* *_r_* = 351.83
Monoclinic, 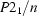

*a* = 6.3171 (13) Å
*b* = 14.535 (3) Å
*c* = 18.169 (4) Å
β = 96.85 (3)°
*V* = 1656.4 (6) Å^3^
*Z* = 4
Mo *K*α radiation
μ = 0.36 mm^−1^
*T* = 293 (2) K
0.15 × 0.09 × 0.07 mm

#### Data collection

Bruker SMART APEX CCD diffractometer
Absorption correction: multi-scan (*SADABS*; Sheldrick, 1996 ▶) *T*_min_ = 0.963, *T*_max_ = 0.971
16788 measured reflections
3788 independent reflections
2287 reflections with *I* > 2σ(*I*)
*R*_int_ = 0.069

#### Refinement

*R*[*F* ^2^ > 2σ(*F* ^2^)] = 0.057
*wR*(*F* ^2^) = 0.129
*S* = 1.04
3788 reflections
217 parameters
H-atom parameters constrained
Δρ_max_ = 0.21 e Å^−3^
Δρ_min_ = −0.31 e Å^−3^

### 

Data collection: *CrystalClear* (Rigaku, 2000 ▶); cell refinement: *CrystalClear*; data reduction: *CrystalStructure* (Rigaku/MSC, 2003 ▶); program(s) used to solve structure: *SHELXS97* (Sheldrick, 2008 ▶); program(s) used to refine structure: *SHELXL97* (Sheldrick, 2008 ▶); molecular graphics: *SHELXTL* (Sheldrick, 2008 ▶); software used to prepare material for publication: *SHELXL97*.

## Supplementary Material

Crystal structure: contains datablocks I, global. DOI: 10.1107/S1600536809003468/cv2506sup1.cif

Structure factors: contains datablocks I. DOI: 10.1107/S1600536809003468/cv2506Isup2.hkl

Additional supplementary materials:  crystallographic information; 3D view; checkCIF report

## Figures and Tables

**Table 1:** Hydrogen-bond geometry (Å, °)

*D*—H⋯*A*	*D*—H	H⋯*A*	*D*⋯*A*	*D*—H⋯*A*
N1—H1*A*⋯O1^i^	0.86	2.54	3.283 (3)	145

Symmetry code: (i) 
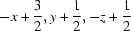
.

**Fig. 1.**
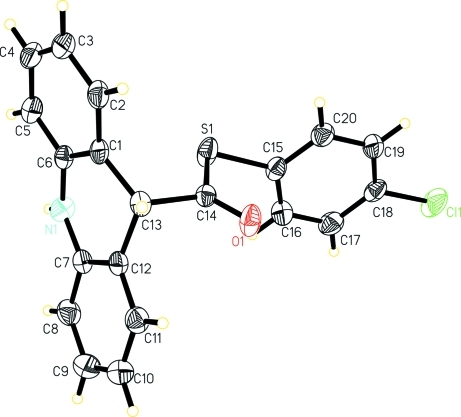
The molecular structure of the title compound with the atom-numbering scheme. Displacement ellipsoids are drawn at the 30% probability level.

## supplementary crystallographic information

### Comment

The title compound, (I), was synthesized from *S*-4-chlorophenyl acridine-9-carbothioate catalyzed by zinc powder and glacial acetic acid using dichlormethane as solvent under the protection of nitrogen at room temperature. (I) is an important intermediate for the synthesis of acridine derivatives which are precursors of practically important chemiluminescent indicators and the chemiluminogenic fragments of chemiluminescent labels (Dodeigne *et al.*, 2000; Ashmore *et al., *2008; Zomer & Jacquemijns, 2001).

In (I) (Fig. 1), the 1,4-dihydropyridine ring (C1/C6/C7/C12/C13/N1) adopts a boat conformation: atoms C1, C6, C7 and C12 are coplanar, with atoms C13 and N1 derivating from the plane by 0.072 (9) and 0.300 (9) Å, respectively. The dihedral angle between the C1-C6 ring and C1/C6/C7/C12 plane is 11.43 (5)°. The dihedral angle between the C7-C12 plane and C1/C6/C7/C12 plane is 13.68 (5)°. In the crystal, weak intermolecular N—H···O hydrogen bonds (Table 1). link the molecules into chains propagated along *b* axis.

### Experimental

All chemicals used(reagent grade) were commercially available. *S*-4-chlorophenyl acridine-9-carbothioate 5.58 g (0.016 mol) was dissolved by dichlormethane, zinc powder 6 g (0.09 mol) and glacial acetic acid 2 mL were added and stirred under the protection of nitrogen at room temperature for 45 min. The mixture was filtered, and evaporated the dissolvent. Colorless crystal of the title compound suitable for X-ray analysis was obtained by recrystallization using dichlormethane. ^1^HMNR(300 MHz, CDCl_3_): 5.22(1*H*, s), 6.80(1*H*, s), 6.81(2*H*, d), 6.94–6.98(2*H*, t), 7.18(2*H*, t), 7.23(2*H*, dd), 7.27(2*H*, dd), 7.30(2*H*,d).

### Refinement

All H atoms were placed in calculated positions (N–H 0.86 Å, C—H 0.93–0.98 Å), and included in the final cycles of refinement using a riding model, with *U*_iso_(H) = 1.2*U*_eq_ of the parent atom.

### Crystal data

**Table:**

C_20_H_14_ClNOS	*F*(000) = 728
*M**_r_* = 351.83	*D*_x_ = 1.411 Mg m^−^^3^
Monoclinic, *P*2_1_/*n*	Mo *K*α radiation, λ = 0.71073 Å
Hall symbol: -P 2yn	Cell parameters from 12319 reflections
*a* = 6.3171 (13) Å	θ = 3.0–27.5°
*b* = 14.535 (3) Å	µ = 0.36 mm^−^^1^
*c* = 18.169 (4) Å	*T* = 293 K
β = 96.85 (3)°	Block, colorless
*V* = 1656.4 (6) Å^3^	0.15 × 0.09 × 0.07 mm
*Z* = 4	

### Data collection

**Table:**

Bruker SMART APEX CCD diffractometer	3788 independent reflections
Radiation source: fine-focus sealed tube	2287 reflections with *I* > 2σ(*I*)
graphite	*R*_int_ = 0.069
ω scans	θ_max_ = 27.5°, θ_min_ = 3.0°
Absorption correction: multi-scan (*SADABS*; Sheldrick, 1996)	*h* = −8→8
*T*_min_ = 0.963, *T*_max_ = 0.971	*k* = −18→18
16788 measured reflections	*l* = −23→23

### Refinement

**Table:**

Refinement on *F*^2^	Primary atom site location: structure-invariant direct methods
Least-squares matrix: full	Secondary atom site location: difference Fourier map
*R*[*F*^2^ > 2σ(*F*^2^)] = 0.057	Hydrogen site location: inferred from neighbouring sites
*wR*(*F*^2^) = 0.129	H-atom parameters constrained
*S* = 1.04	*w* = 1/[σ^2^(*F*_o_^2^) + (0.0388*P*)^2^ + 0.6284*P*] where *P* = (*F*_o_^2^ + 2*F*_c_^2^)/3
3788 reflections	(Δ/σ)_max_ = 0.001
217 parameters	Δρ_max_ = 0.21 e Å^−^^3^
0 restraints	Δρ_min_ = −0.31 e Å^−^^3^

### Special details

**Table:**

Geometry. All e.s.d.'s (except the e.s.d. in the dihedral angle between two l.s. planes) are estimated using the full covariance matrix. The cell e.s.d.'s are taken into account individually in the estimation of e.s.d.'s in distances, angles and torsion angles; correlations between e.s.d.'s in cell parameters are only used when they are defined by crystal symmetry. An approximate (isotropic) treatment of cell e.s.d.'s is used for estimating e.s.d.'s involving l.s. planes.
Refinement. Refinement of *F*^2^ against ALL reflections. The weighted *R*-factor *wR* and goodness of fit *S* are based on *F*^2^, conventional *R*-factors *R* are based on *F*, with *F* set to zero for negative *F*^2^. The threshold expression of *F*^2^ > σ(*F*^2^) is used only for calculating *R*-factors(gt) *etc*. and is not relevant to the choice of reflections for refinement. *R*-factors based on *F*^2^ are statistically about twice as large as those based on *F*, and *R*- factors based on ALL data will be even larger.

### Fractional atomic coordinates and isotropic or equivalent isotropic displacement parameters (Å^2^)

**Table:**

	*x*	*y*	*z*	*U*_iso_*/*U*_eq_	
S1	0.79621 (14)	0.07317 (5)	0.13309 (4)	0.0622 (3)	
Cl1	0.31742 (17)	−0.27324 (6)	−0.00643 (5)	0.0881 (3)	
O1	0.9837 (3)	−0.05520 (12)	0.21991 (11)	0.0601 (6)	
N1	0.7547 (4)	0.24449 (14)	0.27564 (13)	0.0506 (6)	
H1A	0.6458	0.2804	0.2693	0.061*	
C1	1.0780 (4)	0.19172 (17)	0.23159 (14)	0.0408 (6)	
C2	1.2502 (5)	0.21117 (19)	0.19322 (16)	0.0527 (7)	
H2A	1.3570	0.1674	0.1919	0.063*	
C3	1.2669 (5)	0.2938 (2)	0.15705 (17)	0.0630 (9)	
H3A	1.3837	0.3056	0.1318	0.076*	
C4	1.1082 (5)	0.3588 (2)	0.15878 (17)	0.0597 (8)	
H4A	1.1162	0.4141	0.1335	0.072*	
C5	0.9387 (5)	0.34239 (18)	0.19761 (16)	0.0530 (7)	
H5A	0.8337	0.3870	0.1990	0.064*	
C6	0.9226 (4)	0.25965 (17)	0.23493 (15)	0.0419 (6)	
C7	0.7560 (4)	0.17308 (17)	0.32648 (14)	0.0441 (6)	
C8	0.6100 (5)	0.1711 (2)	0.37797 (16)	0.0584 (8)	
H8A	0.5062	0.2166	0.3771	0.070*	
C9	0.6178 (6)	0.1023 (2)	0.43034 (18)	0.0703 (9)	
H9A	0.5190	0.1016	0.4644	0.084*	
C10	0.7702 (6)	0.0346 (2)	0.43274 (17)	0.0660 (9)	
H10A	0.7774	−0.0109	0.4689	0.079*	
C11	0.9126 (5)	0.03501 (19)	0.38071 (16)	0.0534 (7)	
H11A	1.0146	−0.0113	0.3818	0.064*	
C12	0.9071 (4)	0.10270 (16)	0.32687 (14)	0.0412 (6)	
C13	1.0523 (4)	0.09894 (16)	0.26639 (14)	0.0397 (6)	
H13A	1.1931	0.0778	0.2884	0.048*	
C14	0.9580 (4)	0.02581 (17)	0.21057 (14)	0.0392 (6)	
C15	0.6677 (5)	−0.02802 (18)	0.09621 (15)	0.0472 (7)	
C16	0.4798 (5)	−0.0559 (2)	0.12017 (17)	0.0616 (8)	
H16A	0.4246	−0.0231	0.1575	0.074*	
C17	0.3721 (5)	−0.1317 (2)	0.08959 (19)	0.0627 (8)	
H17A	0.2452	−0.1505	0.1061	0.075*	
C18	0.4549 (5)	−0.17925 (19)	0.03436 (16)	0.0525 (7)	
C19	0.6430 (5)	−0.15349 (19)	0.01005 (16)	0.0590 (8)	
H19A	0.6984	−0.1867	−0.0270	0.071*	
C20	0.7491 (5)	−0.07752 (19)	0.04145 (16)	0.0545 (7)	
H20A	0.8773	−0.0595	0.0254	0.065*	

### Atomic displacement parameters (Å^2^)

**Table:**

	*U*^11^	*U*^22^	*U*^33^	*U*^12^	*U*^13^	*U*^23^
S1	0.0851 (6)	0.0375 (4)	0.0563 (5)	−0.0065 (4)	−0.0236 (4)	−0.0005 (3)
Cl1	0.1100 (8)	0.0614 (5)	0.0877 (7)	−0.0439 (5)	−0.0092 (6)	−0.0036 (4)
O1	0.0727 (14)	0.0352 (10)	0.0672 (13)	0.0101 (9)	−0.0133 (11)	−0.0050 (9)
N1	0.0417 (13)	0.0450 (13)	0.0655 (16)	0.0110 (11)	0.0080 (12)	0.0063 (11)
C1	0.0369 (14)	0.0391 (14)	0.0446 (15)	−0.0052 (11)	−0.0031 (12)	−0.0110 (12)
C2	0.0458 (17)	0.0481 (16)	0.0647 (19)	−0.0078 (13)	0.0086 (14)	−0.0177 (14)
C3	0.065 (2)	0.060 (2)	0.067 (2)	−0.0258 (17)	0.0160 (17)	−0.0148 (16)
C4	0.071 (2)	0.0448 (16)	0.063 (2)	−0.0156 (16)	0.0051 (17)	−0.0029 (14)
C5	0.0556 (18)	0.0400 (15)	0.0613 (19)	−0.0007 (13)	−0.0022 (15)	−0.0024 (13)
C6	0.0426 (15)	0.0381 (14)	0.0433 (15)	−0.0049 (12)	−0.0021 (12)	−0.0067 (11)
C7	0.0476 (16)	0.0404 (14)	0.0436 (15)	0.0016 (12)	0.0026 (13)	−0.0078 (12)
C8	0.059 (2)	0.0567 (18)	0.062 (2)	0.0092 (15)	0.0154 (16)	−0.0090 (15)
C9	0.083 (2)	0.072 (2)	0.060 (2)	−0.002 (2)	0.0257 (18)	−0.0017 (18)
C10	0.086 (2)	0.0584 (19)	0.054 (2)	−0.0017 (18)	0.0119 (18)	0.0041 (15)
C11	0.0609 (19)	0.0470 (16)	0.0505 (17)	0.0069 (14)	−0.0002 (15)	−0.0012 (13)
C12	0.0428 (15)	0.0378 (13)	0.0415 (15)	−0.0010 (12)	−0.0014 (12)	−0.0070 (11)
C13	0.0329 (14)	0.0406 (14)	0.0443 (15)	0.0044 (11)	−0.0012 (11)	−0.0061 (11)
C14	0.0372 (14)	0.0370 (14)	0.0434 (15)	0.0003 (11)	0.0050 (11)	−0.0028 (11)
C15	0.0544 (18)	0.0388 (14)	0.0444 (16)	−0.0044 (13)	−0.0102 (13)	0.0012 (12)
C16	0.072 (2)	0.0538 (19)	0.060 (2)	−0.0016 (16)	0.0084 (17)	−0.0086 (15)
C17	0.0531 (19)	0.0609 (19)	0.076 (2)	−0.0119 (16)	0.0141 (17)	0.0053 (17)
C18	0.0607 (19)	0.0430 (15)	0.0500 (17)	−0.0166 (14)	−0.0097 (14)	0.0069 (13)
C19	0.078 (2)	0.0481 (17)	0.0500 (18)	−0.0111 (16)	0.0060 (16)	−0.0104 (14)
C20	0.0545 (19)	0.0500 (17)	0.0586 (18)	−0.0112 (14)	0.0055 (15)	−0.0007 (14)

### Geometric parameters (Å, °)

**Table:**

S1—C15	1.772 (3)	C8—H8A	0.9300
S1—C14	1.777 (3)	C9—C10	1.373 (4)
Cl1—C18	1.737 (3)	C9—H9A	0.9300
O1—C14	1.198 (3)	C10—C11	1.380 (4)
N1—C6	1.381 (3)	C10—H10A	0.9300
N1—C7	1.389 (3)	C11—C12	1.385 (4)
N1—H1A	0.8600	C11—H11A	0.9300
C1—C2	1.389 (4)	C12—C13	1.514 (4)
C1—C6	1.399 (4)	C13—C14	1.539 (3)
C1—C13	1.506 (3)	C13—H13A	0.9800
C2—C3	1.380 (4)	C15—C16	1.373 (4)
C2—H2A	0.9300	C15—C20	1.376 (4)
C3—C4	1.380 (4)	C16—C17	1.378 (4)
C3—H3A	0.9300	C16—H16A	0.9300
C4—C5	1.371 (4)	C17—C18	1.372 (4)
C4—H4A	0.9300	C17—H17A	0.9300
C5—C6	1.390 (4)	C18—C19	1.368 (4)
C5—H5A	0.9300	C19—C20	1.380 (4)
C7—C8	1.391 (4)	C19—H19A	0.9300
C7—C12	1.398 (3)	C20—H20A	0.9300
C8—C9	1.378 (4)		
			
C15—S1—C14	99.92 (12)	C10—C11—C12	121.6 (3)
C6—N1—C7	122.1 (2)	C10—C11—H11A	119.2
C6—N1—H1A	119.0	C12—C11—H11A	119.2
C7—N1—H1A	119.0	C11—C12—C7	118.9 (3)
C2—C1—C6	118.3 (2)	C11—C12—C13	121.4 (2)
C2—C1—C13	121.5 (2)	C7—C12—C13	119.7 (2)
C6—C1—C13	120.2 (2)	C1—C13—C12	112.2 (2)
C3—C2—C1	121.7 (3)	C1—C13—C14	113.2 (2)
C3—C2—H2A	119.2	C12—C13—C14	106.4 (2)
C1—C2—H2A	119.2	C1—C13—H13A	108.3
C2—C3—C4	119.2 (3)	C12—C13—H13A	108.3
C2—C3—H3A	120.4	C14—C13—H13A	108.3
C4—C3—H3A	120.4	O1—C14—C13	123.4 (2)
C5—C4—C3	120.4 (3)	O1—C14—S1	123.3 (2)
C5—C4—H4A	119.8	C13—C14—S1	113.29 (17)
C3—C4—H4A	119.8	C16—C15—C20	119.1 (3)
C4—C5—C6	120.6 (3)	C16—C15—S1	119.9 (2)
C4—C5—H5A	119.7	C20—C15—S1	120.9 (2)
C6—C5—H5A	119.7	C15—C16—C17	120.8 (3)
N1—C6—C5	120.3 (2)	C15—C16—H16A	119.6
N1—C6—C1	119.9 (2)	C17—C16—H16A	119.6
C5—C6—C1	119.8 (3)	C18—C17—C16	119.0 (3)
N1—C7—C8	120.7 (2)	C18—C17—H17A	120.5
N1—C7—C12	120.0 (2)	C16—C17—H17A	120.5
C8—C7—C12	119.3 (3)	C19—C18—C17	121.3 (3)
C9—C8—C7	120.5 (3)	C19—C18—Cl1	119.1 (2)
C9—C8—H8A	119.8	C17—C18—Cl1	119.6 (2)
C7—C8—H8A	119.8	C18—C19—C20	118.9 (3)
C10—C9—C8	120.6 (3)	C18—C19—H19A	120.5
C10—C9—H9A	119.7	C20—C19—H19A	120.5
C8—C9—H9A	119.7	C15—C20—C19	120.8 (3)
C9—C10—C11	119.1 (3)	C15—C20—H20A	119.6
C9—C10—H10A	120.4	C19—C20—H20A	119.6
C11—C10—H10A	120.4		
			
C6—C1—C2—C3	−2.0 (4)	C2—C1—C13—C12	159.2 (2)
C13—C1—C2—C3	175.6 (2)	C6—C1—C13—C12	−23.3 (3)
C1—C2—C3—C4	−0.2 (4)	C2—C1—C13—C14	−80.3 (3)
C2—C3—C4—C5	1.7 (4)	C6—C1—C13—C14	97.2 (3)
C3—C4—C5—C6	−0.9 (4)	C11—C12—C13—C1	−159.6 (2)
C7—N1—C6—C5	−165.4 (2)	C7—C12—C13—C1	23.8 (3)
C7—N1—C6—C1	14.2 (4)	C11—C12—C13—C14	76.1 (3)
C4—C5—C6—N1	178.2 (3)	C7—C12—C13—C14	−100.5 (3)
C4—C5—C6—C1	−1.4 (4)	C1—C13—C14—O1	156.3 (3)
C2—C1—C6—N1	−176.8 (2)	C12—C13—C14—O1	−80.0 (3)
C13—C1—C6—N1	5.6 (4)	C1—C13—C14—S1	−26.6 (3)
C2—C1—C6—C5	2.8 (4)	C12—C13—C14—S1	97.1 (2)
C13—C1—C6—C5	−174.8 (2)	C15—S1—C14—O1	11.1 (3)
C6—N1—C7—C8	165.7 (3)	C15—S1—C14—C13	−166.00 (19)
C6—N1—C7—C12	−13.5 (4)	C14—S1—C15—C16	89.1 (2)
N1—C7—C8—C9	−177.2 (3)	C14—S1—C15—C20	−93.6 (2)
C12—C7—C8—C9	2.0 (4)	C20—C15—C16—C17	−0.6 (4)
C7—C8—C9—C10	0.2 (5)	S1—C15—C16—C17	176.8 (2)
C8—C9—C10—C11	−1.7 (5)	C15—C16—C17—C18	−0.3 (5)
C9—C10—C11—C12	0.9 (5)	C16—C17—C18—C19	1.0 (5)
C10—C11—C12—C7	1.3 (4)	C16—C17—C18—Cl1	−178.3 (2)
C10—C11—C12—C13	−175.3 (3)	C17—C18—C19—C20	−0.8 (4)
N1—C7—C12—C11	176.5 (2)	Cl1—C18—C19—C20	178.5 (2)
C8—C7—C12—C11	−2.7 (4)	C16—C15—C20—C19	0.8 (4)
N1—C7—C12—C13	−6.9 (4)	S1—C15—C20—C19	−176.6 (2)
C8—C7—C12—C13	173.9 (2)	C18—C19—C20—C15	−0.2 (4)

### Hydrogen-bond geometry (Å, °)

**Table:**

*D*—H···*A*	*D*—H	H···*A*	*D*···*A*	*D*—H···*A*
N1—H1A···O1^i^	0.86	2.54	3.283 (3)	145

Symmetry codes: (i) −*x*+3/2, *y*+1/2, −*z*+1/2.
